# Steady‐state free precession for T_2_ * relaxometry: All echoes in every readout with k‐space aliasing

**DOI:** 10.1002/mrm.30590

**Published:** 2025-06-02

**Authors:** Peter J. Lally, Yifei Jin, Zimu Huo, Coraline Beitone, Mark Chiew, Paul M. Matthews, Karla L. Miller, Neal K. Bangerter

**Affiliations:** ^1^ Department of Bioengineering Imperial College London London UK; ^2^ Centre for Care Research and Technology UK Dementia Research Institute London UK; ^3^ London Collaborative Ultra‐high field System (LoCUS) London UK; ^4^ Medical Biophysics University of Toronto Toronto Ontario Canada; ^5^ Physical Sciences Sunnybrook Research Institute Toronto Ontario Canada; ^6^ Department of Brain Sciences Imperial College London London UK; ^7^ UK Dementia Research Institute at Imperial London UK; ^8^ Oxford Centre for Integrative Neuroimaging University of Oxford Oxford UK; ^9^ Department of Electrical and Computer Engineering Boise State University Boise Idaho USA

**Keywords:** FLASH, OSSI, SSFP, T_2_*

## Abstract

**Purpose:**

Multi‐echo gradient echo imaging is useful for a range of applications including relaxometry, susceptibility mapping, and quantifying relative proportions of fat and water. This relies primarily on long‐TR multi‐echo gradient echo sequences (FLASH), which by design isolate one signal component (i.e., echo) at a time per readout. In this work, we propose an alternative strategy that simultaneously measures all signal components at once in every readout event with an N‐periodic SSFP sequence. Essentially, we Fourier encode the signals into an “F‐k space” similar to the “TE‐k space” of a multi‐echo gradient echo acquisition. This enables an efficient, short‐TR relaxometry experiment where signals benefit from averaging effects over multiple excitations.

**Theory and Methods:**

In the presented approach, multiple echoes are recorded simultaneously and separated by their differing phase evolution over multiple TRs. At low flip angles the relative echo amplitudes and phases are equivalent to those acquired sequentially from a multi‐echo FLASH, in terms of both T_2_* weighting and spatial phase distributions. The two approaches were compared for the example of R_2_* relaxometry in a phantom and in human volunteers.

**Results:**

The proposed approach shows close agreement in R_2_* estimation with multi‐echo FLASH, with the advantage of more rapid temporal sampling.

**Conclusion:**

The proposed approach is a promising alternative to other relaxometry approaches, by measuring signals from multiple echo pathways simultaneously and separating them based on a simple analytical model.

## 1 INTRODUCTION

T_2_*‐weighted gradient echo techniques are widely used for quantitative and functional imaging, and most commonly examine magnetization that is excited at the RF pulse and evolves until the TE, or across the series of TEs in the case of multi‐echo techniques. However, the need for a long TR (with TR > TE) limits temporal resolution for spatial encoding. This in turn limits the ability to directly measure rapid dynamic processes of interest (e.g., neural activity)^1^, ^2^, ^3^ as well as characterizing transient nuisances (e.g., flow, pulsation) for their removal.^4^, ^5^ There is, therefore, a need to develop approaches that maintain sufficient T_2_*‐sensitivity and SNR while also improving sampling efficiency.

Echo‐shifted T_2_*‐weighted imaging approaches have long been proposed to enable a short TR for efficient spatial encoding and high SNR efficiency.^6^ In echo‐shifted techniques the effective TE is longer than the TR, enabling both rapid temporal sampling of whole volumes and sufficient T_2_*‐weighting to maintain sensitivity to the contrast of interest. This concept has been applied in different forms and with various readout schemes to accelerate BOLD fMRI.^7^, ^8^, ^9^, ^10^, ^11^

More recently oscillating steady‐state imaging (OSSI) has been proposed as another short‐TR approach that achieves strongly T_2_*‐weighted images and SNR efficiency superior to other gradient echo techniques for fMRI.^12^ In this approach, a pseudo‐steady state is reached in which the measured magnetization fluctuates from TR‐to‐TR, in a regular periodic pattern. Although the OSSI signal has been shown to have a strong T_2_* dependence, it lacks an analytical framework to support experimental design.

In this work, we examine the fluctuating magnetization behavior in OSSI and derive an analytical form that links its TR‐to‐TR signal changes to the constructive and destructive interference of multiple T_2_*‐weighted signal pathways. We demonstrate a novel approach to separate the fluctuating signal into its constituent pathways, showing that OSSI is not just a T_2_*‐weighted approach, but also can be adapted as a relaxometry technique. We highlight deviations of the derived signal components from a “pure” T_2_* decay curve and propose a modified acquisition approach that restores T_2_*‐dependence. We compare these approaches to equivalent multi‐echo T_2_* FLASH acquisitions in simulations and experiments.

## 2 THEORY

In this section, we first outline the analytical form for the N‐periodic fluctuations in the steady‐state magnetization observed under a series of RF pulses with quadratically increasing phase. We then show how this analytical form can be applied to isolate different T_2_*‐weighted signal components from the fluctuating signal time series. Finally, we show how the acquisition approach can be adapted with a small amount of gradient spoiling to isolate a subset of the T_2_*‐weighted signal components that can be used for a relaxometry experiment.

### 2.1 Quadratic RF phase cycling and N‐periodic steady states

Quadratic RF phase cycling is widely used in gradient echo sequences, with the *n*th RF pulse having phase $\varphi_{n}=\varphi_{\text{quad}}n^{2}/2$, where $\varphi_{\text{quad}}$ is the chosen quadratic phase cycling increment. This has the effect of a linear change in the center frequency in successive TRs.^13^

**FIGURE 1:**
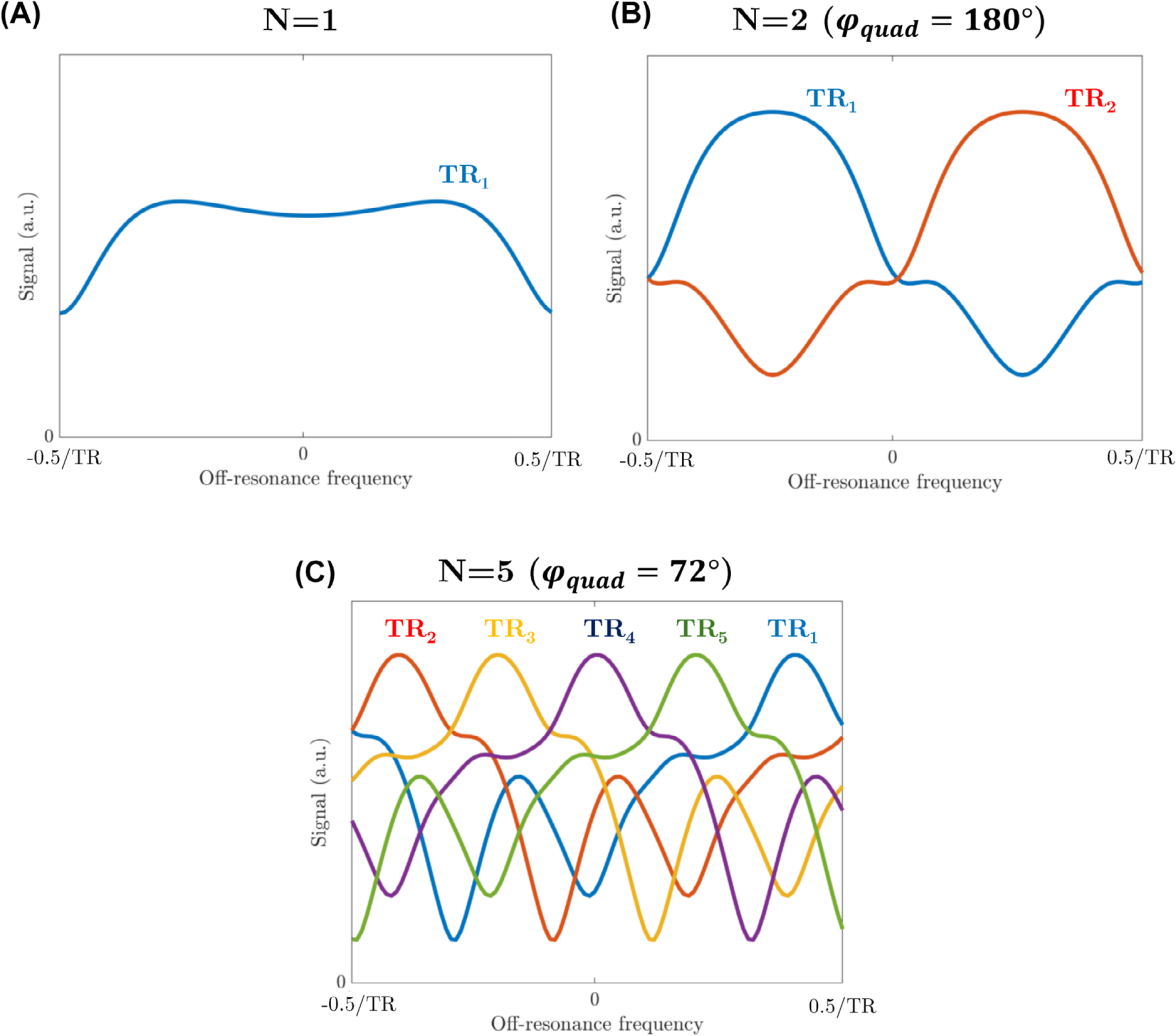
Per‐TR off‐resonance profiles for different N‐periodic RF phase cycling schemes. (A) For *N* = 1 the familiar off‐resonance profile for balanced SSFP is obtained, and (B) *N* = 2 (FEMR^15^) provides differing off‐resonance profiles for even and odd TRs. (C) This can be extended to any *N*, shown here for *N* = 5.

An important feature of quadratic phase increments is that they are N‐periodic.^14^ Where *N* = 360° $/\varphi_{\text{quad}}$ and N is a positive integer, the steady‐state magnetization behavior repeats after every Nth RF pulse. Figure 1 demonstrates the magnetization behavior for different N‐periodic experiments, where *N* = 5, 2, and 1 (where *N* = 2 corresponds to fluctuating equilibrium MRI (FEMR),^15^ and *N* = 1 corresponds to balanced SSFP [bSSFP]). N‐periodic steady states are, therefore, periodic in both off‐resonance frequency (i.e., over the range of 1/TR) and time (i.e., over N TRs). In RF‐spoiled sequences, N is typically chosen with N ∉ℕ to avoid this periodic behavior, and $\varphi_{\text{quad}}$ is empirically tuned to minimize T_2_ contrast (e.g., 117° or 50°).^16^

### 2.2 Phase dependency in quadratic RF phase cycled sequences

At low flip angles (α<30°) we can assume each RF pulse causes minimal conversion of transverse magnetization into longitudinal magnetization (i.e., avoiding stimulating echoes) and the magnitude M_F_ of each signal component in an RF‐spoiled sequence can then be described as follows^14^:

(1) $$M_{F}\left(\mathrm{TE}\right)=e^{-\left(\mathrm{TE}+F.\mathrm{TR}\right)/T_{2}^{*}}\sin\alpha \frac{1-e^{-\mathrm{TR}/T_{1}}}{1-\left(\cos\alpha \right)e^{-\mathrm{TR}/T_{1}}},$$

where F is an integer (F∈ℤ) denoting the order of the configuration state (“F‐state” from the extended phase graph formalism,^17^ indexing the echo pathway that generates the signal) and α is the flip angle. A more general signal model that accounts for higher flip angle RF pulses is also available,^14^ but not explored in this work.

Incorporating local off‐resonance (ω), the signal from each F‐state at TE is given by:

(2) $$S_{F}\left(\mathrm{TE}\right)=M_{F}\left(\mathrm{TE}\right)e^{i\omega \left(\mathrm{TE}+F.\mathrm{TR}\right)}.$$

The measured signal is then the sum across all F‐states after incorporating both linear ($\varphi_{n}=\varphi_{\mathrm{lin}}n$) and quadratic ($\varphi_{n}=\varphi_{\text{quad}}n^{2}/2$) RF phase cycling increments, where *n* is the index of the RF pulse:

(3) $$S\left(\mathrm{TE},n\right)=\sum_{F}S_{F}\left(\mathrm{TE}\right)e^{\text{iF}\left(n\varphi_{\text{quad}}+\varphi_{\mathrm{lin}}\right)},$$

(4) $$=e^{\text{iωTE}}\sum_{F}M_{F}\left(\mathrm{TE}\right)e^{\text{iF}\left(\varphi_{\mathrm{lin}}+\omega \mathrm{TR}\right)}e^{\mathrm{inF}\varphi_{\text{quad}}}.$$

The phase terms in Eq. (3) with respect to *F* can be grouped into: (1) a global term, $e^{\text{iωTE}}$; (2) a constant linear term, $e^{iF\left(\varphi_{\mathrm{lin}}+\omega \mathrm{TR}\right)}$; and (3) an *n*‐dependent linear term in F, $e^{i\mathrm{nF}\varphi_{\text{quad}}}$, which causes a per‐TR phase change. From here onward, we assume $\varphi_{\mathrm{lin}}$ = 0° for simplicity, and focus on the quadratic term $\varphi_{\text{quad}}$, which causes the per‐TR phase change.

Intuitively, Eq. (4) is a discrete Fourier decomposition that maps the fluctuating signal across a series of *N* TRs (index n) to its constituent F‐state components (equivalent to Fourier coefficients). This phase modulation on the *F*th state in the *n*th TR can also be expressed as a matrix operator $\Psi_{\mathrm{nF}}$:

(5) $$\Psi_{\mathrm{nF}}=e^{\mathrm{inF}\varphi_{\text{quad}}},$$

which gives *N* linearly independent rows for an *N*‐periodic acquisition (i.e., $\varphi_{\text{quad}}$ = 360°/N), describing the phase evolution of each F‐state. Provided there are fewer F‐state components in the signal than measurements *N* then each row of $\Psi_{\mathrm{nF}}$ forms a complete orthogonal basis to decompose the measured signal into its constituent pathways $S_{F}$. Inverting $\Psi_{\mathrm{nF}}$ then separates the per‐TR signal into its different F‐state signal components, which are themselves measured and averaged over *N* acquisitions. This approach is similar to the formalism introduced by Zur et al.^18^ for linear RF phase cycling, but instead applies this to N‐periodic sequences to enable more predictable signal separation.

### 2.3 Problems with F‐states > N

If the number of F‐state signal components in each measurement is greater than the number of measurements *N*, then Eq. (5) will result in an incomplete basis: each measured F‐state signal component S_F_ will contain contributions from S_F‐N_, S_F‐2N_, … and S_F+N_, S_F+2N_, … components.

In OSSI, a bSSFP acquisition is used with quadratic RF spoiling, which means that all F‐state signal components in the magnetization contribute to each measured signal.^12^ Examining Eq. (1), it follows that the measured signal is a sum of many different T_2_* weighted signal components.

If there was a way of restricting the number of F‐states to be less than N, then it would be possible to perform T_2_* relaxometry with OSSI using (Eqs. 1 and 4). One way of achieving this would be to set N or TR to be long, such that N.TR ≫ T_2_*, and only the first N F‐state signals are detectable above noise.

However, we can also apply an additional spoiler gradient in the pulse sequence to effectively isolate a series of F‐states. We call this approach “k‐space aliasing.”

### 2.4 Choosing a subset of F‐states with k‐space aliasing

**FIGURE 2:**
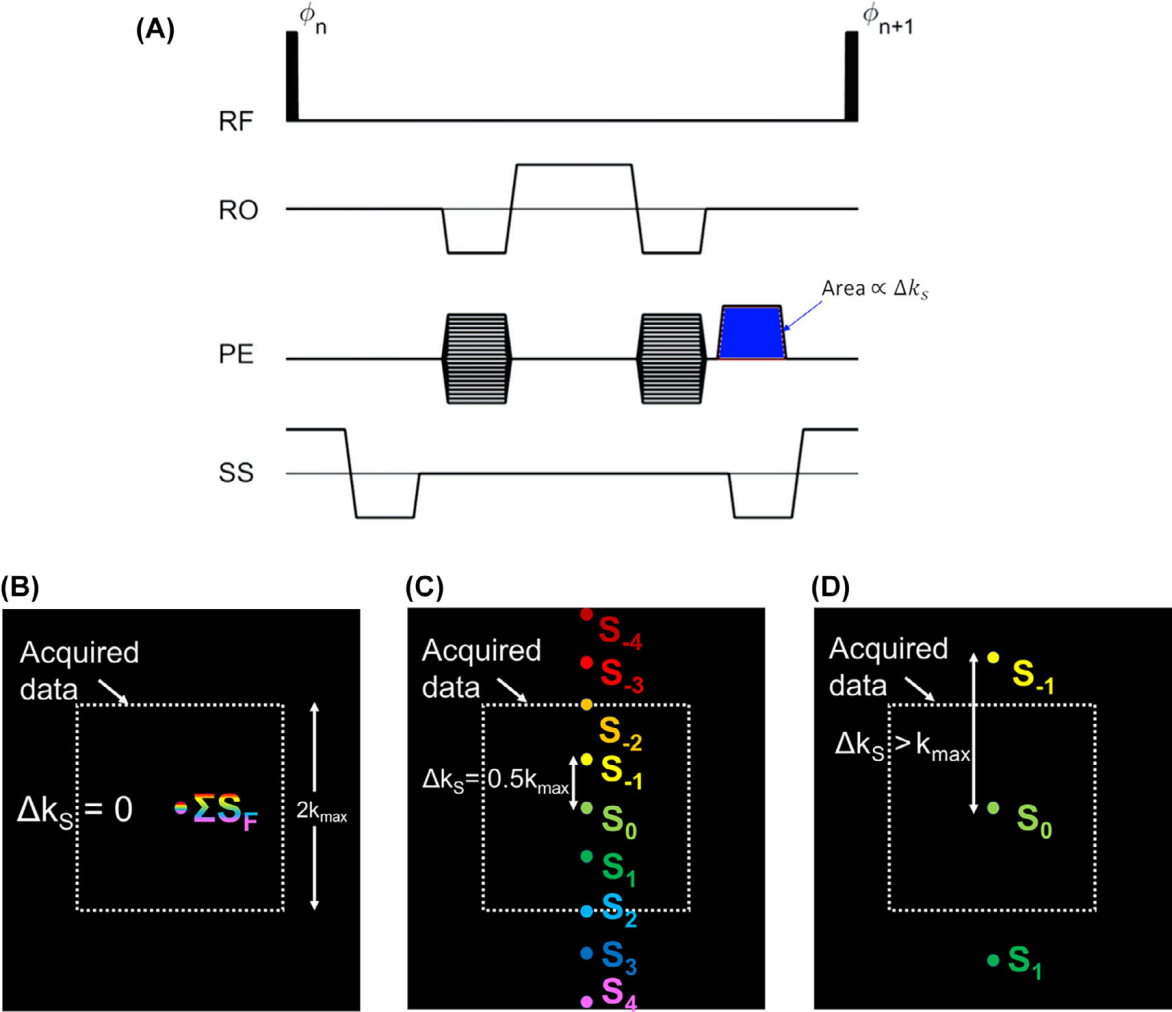
(A) Pulse sequence diagram for an SSFP acquisition, including a single unbalanced spoiler gradient (blue), which determines the orientation and spacing of k‐space aliasing, here shown along the phase encoding direction. The phase of the nth RF pulse is denoted by φ_n_. (B) With no spoiler gradient (as in oscillating steady‐state imaging [OSSI]), all F‐state signals (S_F_) are superimposed at the center of k‐space. (C) For a larger spoiler gradient, F‐state signals are separated by a larger amount in k‐space. (D) For separation larger than the half‐width of the acquired k‐space (*k*_max_) this is typically considered to be gradient spoiled.

If we add an additional, unbalanced spoiler gradient within the TR (shown in blue in Figure 2A), the signal components from different F‐states can be shifted to specific locations in k‐space. Without loss of generality, we assume that this spoiler gradient is the same in every TR. The shifting of successive F‐states, $∆k_{s}$, is given by the time integral of the unbalanced spoiler gradient $G_{s}$ over the TR:

(6) $$∆k_{s}=\bar{\gamma }\int_{0}^{\mathrm{TR}}G_{s}\mathrm{dt},$$

where $\bar{\gamma }$ is the normalized gyromagnetic ratio, and the orientation G_s_ determines the orientation of the F‐state shifts in k‐space. Figure 2B–D illustrates different scenarios where the amount of unbalanced gradient spoiling is varied along a single axis, giving rise to different shifts of F‐state components within the acquired k‐space. Where all gradients are balanced as in OSSI (Figure 2B) all F‐state components are superimposed at the center of k‐space. With a large unbalanced gradient in the phase encoding direction (depicted as up‐down in Figure 2D) one F‐state component predominantly contributes to the measured signal, describing a gradient‐spoiled regime (however, it is important to note that the shifted components still contribute high frequency signals and can be used for super‐resolution^19^, ^20^). In the intermediate cases (Figure 2C), we can measure substantial signal from several F‐state signals simultaneously within our acquired data (“k‐space aliasing”) and can adjust the amount of gradient spoiling to control the number of signals and their spacing. We could also offset the phase encoding scheme to choose which F‐state appears at the center of the measured k‐space.

For a given *N* the simplest acquisition scheme comprises a single period of build up to the steady state, followed by *N* acquisitions at the first phase encoding line, before repeating this for further phase encoding lines in a linear order.

**FIGURE 3:**
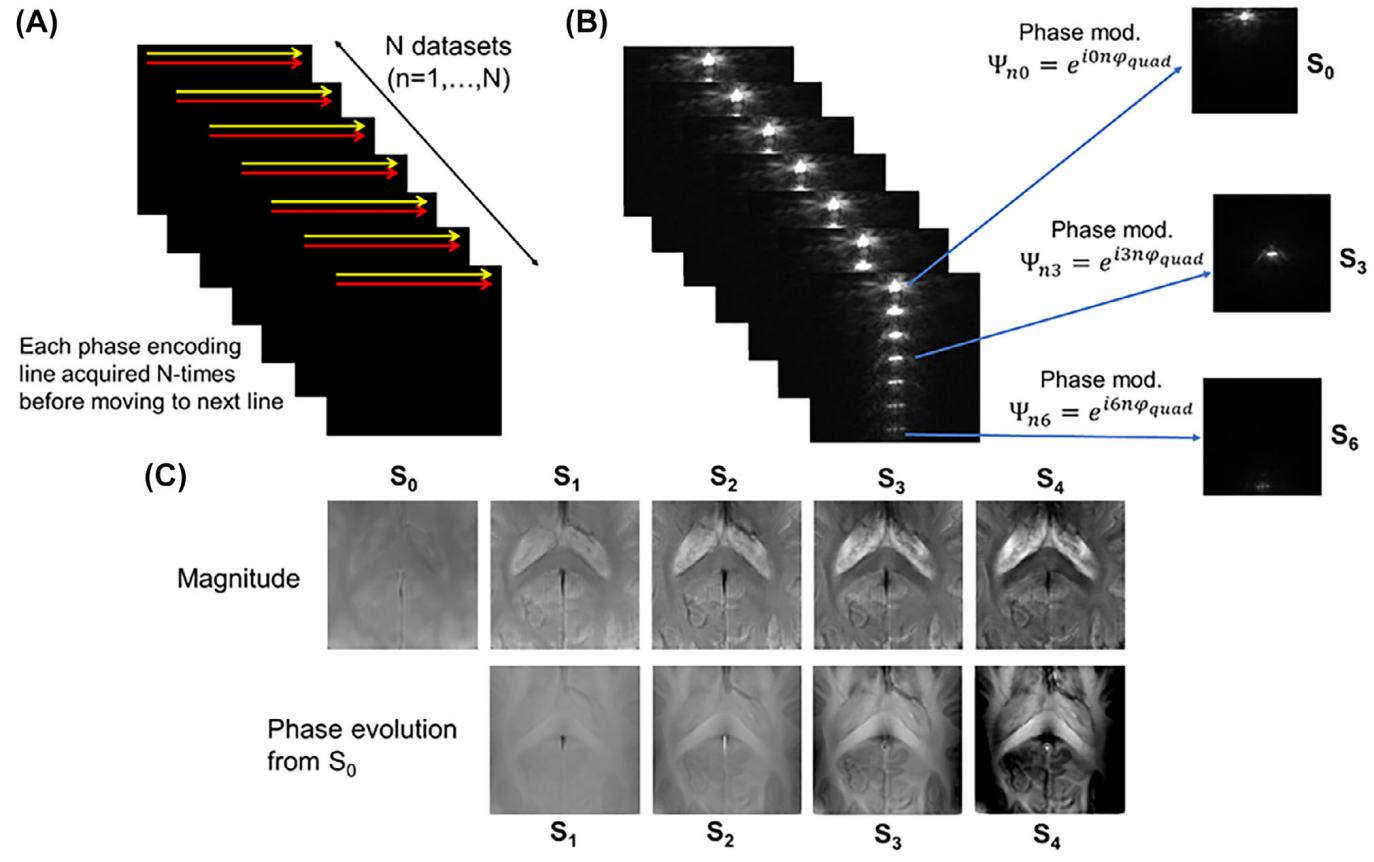
Schematic of the acquisition and reconstruction scheme. (A) A simple acquisition scheme is used with an inner loop over N TRs, and an outer loop of linear phase encoding. (B) Using Eq. (5), each signal component (S_0_–S_6_ here) can be extracted by its known phase evolution over N TRs. (C) The magnitude and phase content of each signal component, resembling that obtained from a multi‐echo FLASH experiment.

In this work, this scheme is adapted as a T_2_* relaxometry experiment as shown in Figure 3, where only the signal magnitude from positive F‐states is considered. The signal magnitude from higher order F‐states is more heavily T_2_* weighted, and the phase is more susceptibility weighted (reflected in the F.TR terms in Eqs. [1] and [2], Figure 3). We describe this proposed short‐TR approach as k‐space‐aliased FLASH (kaFLASH). The F‐state signals S_F_ have relative weightings equivalent to the echo signals obtained from a long‐TR multi‐echo FLASH sequence with (1) a first TE equal to the kaFLASH TE; and (2) an echo spacing equal to the kaFLASH TR.

Given a resolution and FOV, one must choose the amount of gradient spoiling to ensure *M* different T_2_* weighted contrasts can be reconstructed. To recover a series of *M* signal components, one should, therefore, set $∆k_{s}<2k_{\max}/M$, where *k*_max_ is the half‐width of k‐space, to ensure that the highest order F‐states are still captured at the edge of k‐space. Additionally, the central F‐state signal in k‐space should be chosen as the central signal component in the range *M*. In this work, we measure *M* = 7 components (S_0_–S_6_) and so have added an offset to the phase encoding gradients to ensure S_3_ appears at the center of the acquired k‐space (Figure 3B).

### 2.5 SNR efficiency

In terms of SNR, the proposed approach is a compromise between the effect of *N* signal averages and the asymmetric sampling of each signal component. Signals that are located at the center of k‐space are sampled symmetrically (i.e., S_0_ in Figure 2C,D, S_3_ in Figure 3B), whereas other signals that have been shifted in k‐space because of the gradient spoiler are sampled asymmetrically (i.e., all signal components apart from S_0_ in Figure 2C,D or S_3_ in Figure 3B). This results in a relative SNR penalty because of the need for partial Fourier completion of the same symmetric range of spatial frequencies as the central component for fair comparison at matched spatial resolution.

The SNR for each shifted F‐state signal component after partial Fourier reconstruction can then be described as:

(7) $$\mathrm{SNR}\propto \sqrt{1-\left|F_{\text{shifted}}-F_{\text{central}}\right|\frac{∆k_{s}}{2k_{\max}}}\cdot \sqrt{N},$$

where $F_{\text{shifted}}$ and $F_{\text{central}}$ are integers denoting the F‐state indices of the shifted and central components respectively. This simply reflects the fraction of signal acquired, ranging from $\sqrt{0.5}$ (for a signal located at *k*_max_) to 1 (for a signal positioned at the center of the acquired k‐space), and the averaging across the series of measurements, denoted by $\sqrt{N}$. In the case of OSSI, all components are sampled symmetrically, and so there is no SNR penalty.

## 3 METHODS

### 3.1 Phantom study

The proposed kaFLASH sequence was implemented on a 3 T Siemens MAGNETOM Verio with a 32‐channel head coil. $\varphi_{\text{quad}}$ and $∆k_{s}$ were varied to give 5‐periodic ($\varphi_{\text{quad}}$ = 72°, $∆k_{s}$ = 40 lines), 6‐periodic ($\varphi_{\text{quad}}$ = 60°, $∆k_{s}$ = 40 lines), 7‐periodic ($\varphi_{\text{quad}}$ = 51.4°, $∆k_{s}$ = 34 lines), and 12‐periodic ($\varphi_{\text{quad}}$ = 30°, $∆k_{s}$ = 20 lines) experiments in a single sagittal 2D slice across the T_2_ sphere section of a National institute of Standards and Technology / International Society for Magnetic Resonance in Medicine MRI system phantom, with FOV = 256 × 256 × 5 mm^3^, matrix = 256 × 256 × 1, TR = 6.0 ms, TE = 3.0 ms, flip angle = 10°, bandwidth = 528 Hz/pix. A single steady‐state build‐up period of 30 s (kept conservatively long to account for the low relaxivity of some compartments the phantom) was included at the start of each acquisition (total scan times of 7.7 s [*N* = 5], 9.2 s [*N* = 6], 10.8 s [*N* = 7], and 18.4 s [*N* = 12] excluding steady‐state build‐up).

To evaluate the accuracy of T_2_* measurements with the proposed approach in the different spheres, this was compared with a vendor‐supplied multi‐echo FLASH, with matched FOV and matrix size, matched T_2_* weightings for each echo (TE_1_ = 3 ms, ∆ TE = 6 ms), and matched bandwidth, with TR = 100 ms and flip angle = 90°. In all cases, the first five echoes were fit to a monoexponential R_2_* decay function, and R_2_* in each sphere compared between techniques via Bland–Altman analysis, assuming multi‐echo FLASH as the ground truth measure. Phantom data and MATLAB analysis scripts are provided at https://github.com/petelally/nperiodic_analysis.

### 3.2 Human studies

Healthy volunteer brain studies were conducted on a 7 T Siemens MAGNETOM Terra with a 1T×/32R× head coil (Nova Medical). These were approved by the local research ethics committee, with all participants giving prior informed consent.

#### 3.2.1 SNR efficiency comparisons

The proposed kaFLASH sequence was implemented with a 12‐periodic experiment ($\varphi_{\text{quad}}$ = 30°) on a single 2D axial slice, with FOV = 256 × 256 × 5 mm^3^, matrix = 256 × 256 × 1, TR = 8.0 ms, TE = 4.0 ms, and bandwidth = 488 Hz/pix. The gradient spoiling at the end of the TR was set to $∆k_{s}$ = 35 phase encoding lines, with S_3_ at the center of k‐space to allow the direct measurement of 7 F‐states (S_0_–S_6_). A single steady‐state build‐up period of 8 s was included at the start of the sequence, resulting in a total acquisition time of 33 s. This was repeated with flip angles of 1°, 5°, 10°, 15°, and 20° in a single healthy volunteer. The OSSI sequence was implemented as above for kaFLASH, but with a 10‐periodic experiment ($\varphi_{\text{quad}}$ = 36°) and without gradient spoiling, with an acquisition time of 28 s including 8 s to allow for steady‐state build‐up.

The kaFLASH acquisition was compared to a vendor supplied multi‐echo FLASH sequence with a longer TR (= 55 ms), 7 matched TEs (= 4 to 52 ms, ΔTE = 8 ms), bandwidth = 500 Hz/pix, and with matched FOV and resolution, giving a total acquisition time of 14 s. This was repeated with flip angles of 1°, 5°, 10°, 15°, 20°, 25°, 30°, 35°, 40°, 45°, and 50°. The OSSI acquisition was compared to the same multi‐echo FLASH sequence, but with matched TRs and TEs (TR = 79 ms, 10 matched TEs from 4 to 76 ms, ΔTE = 8 ms) and repeated with the same range of flip angles (1°–50°).

Relative SNR efficiency was calculated from the S_3_ kaFLASH image (that was symmetrically sampled and coil‐combined via root sum‐of‐squares) by comparison to a region of background noise calculated from a single coil of the raw data (.dat) files obtained from each acquisition, and normalized by the square root of the acquisition time. For the FLASH and OSSI sequences, the echo with an equivalent TE to the S_3_ kaFLASH signal was selected for SNR efficiency measurement, using the same procedure. In all calculations, the absolute SNR is overestimated, because there is a noise contribution from multiple coil elements in the root sum‐of‐squares image.

#### 3.2.2 Brainstem imaging comparisons

In a second healthy volunteer, we conducted a 3D experiment covering a 2‐cm axial section of the brainstem, again with $\varphi_{\text{quad}}$ = 30° to give a 12‐periodic experiment, FOV = 256 × 256 × 20 mm^3^, matrix = 256 × 256 × 20, TR = 6.0 ms, TE = 3.0 ms, bandwidth = 488 Hz/pix, and flip angle = 10°. The gradient spoiling at the end of the TR was set to $∆k_{s}$ = 35 phase encoding lines in‐plane (i.e., right–left direction), again with S_3_ at the center of k‐space to allow the direct measurement of 7 F‐states (S_0_–S_6_). A single steady‐state build‐up period of 6 s was included at the start of the sequence, resulting in a total acquisition time of 6 min 15 s. This was then retrospectively sub‐sampled (every second TR discarded) to simulate an *N* = 6 acquisition of 3 min 11 s. Importantly, this is different steady state than that produced by a prospective N = 6 acquisition (i.e., the overall magnitude of the signal components will differ), but the phase evolution of each signal component is equivalent.

This was acquired along with a vendor supplied multi‐echo FLASH sequence with a longer TR (= 42 ms), 7 matched TEs (= 3 to 39 ms, ΔTE = 6 ms), bandwidth = 490 Hz/pix, and with matched FOV and resolution, giving an acquisition time of 3 min 35 s.

In a third healthy volunteer, we conducted further kaFLASH experiments at 0.7 mm isotropic resolution to cover a ˜2 cm axial section of the midbrain. S_3_ was at the center of k‐space and the gradient spoiling adjusted to allow the direct measurement of 7 F‐states (S_0_–S_6_).

Acquisition details: $\varphi_{\text{quad}}$ = 30°, FOV = 256 × 256 × 21 mm^3^, matrix = 366 × 366 × 30, TR = 6.4 ms, TE = 3.2 ms, bandwidth = 488 Hz/pix, flip angle = 10°, $∆k_{s}$ = 50 phase encoding lines in‐plane (i.e., right–left direction), and an initial steady‐state build‐up period of 6.4 s, resulting in a total acquisition time of 14 min 10 s. This was also retrospectively subsampled to obtain an *N* = 6 acquisition with an effective acquisition time of 7 min 2 s.

### 3.3 Image reconstruction

In all cases, reconstruction was performed by first computing the isolated F‐state signal components via linear combinations of the acquired k‐space data over the *N* TRs for each element of the receiver coil (i.e., demodulating the acquired data by solving Eq. 5):

(8) $$S_{F}=\sum_{n=1}^{N}S_{n}e^{-\mathrm{inF}\varphi_{\text{quad}}}.$$

Following this, a partial Fourier correction was applied with a projection onto convex sets algorithm, and the individual coil images combined via root sum‐of‐squares. R_2_* maps were fit via non‐linear least squares.

## 4 RESULTS

### 4.1 Phantom study

**FIGURE 4:**
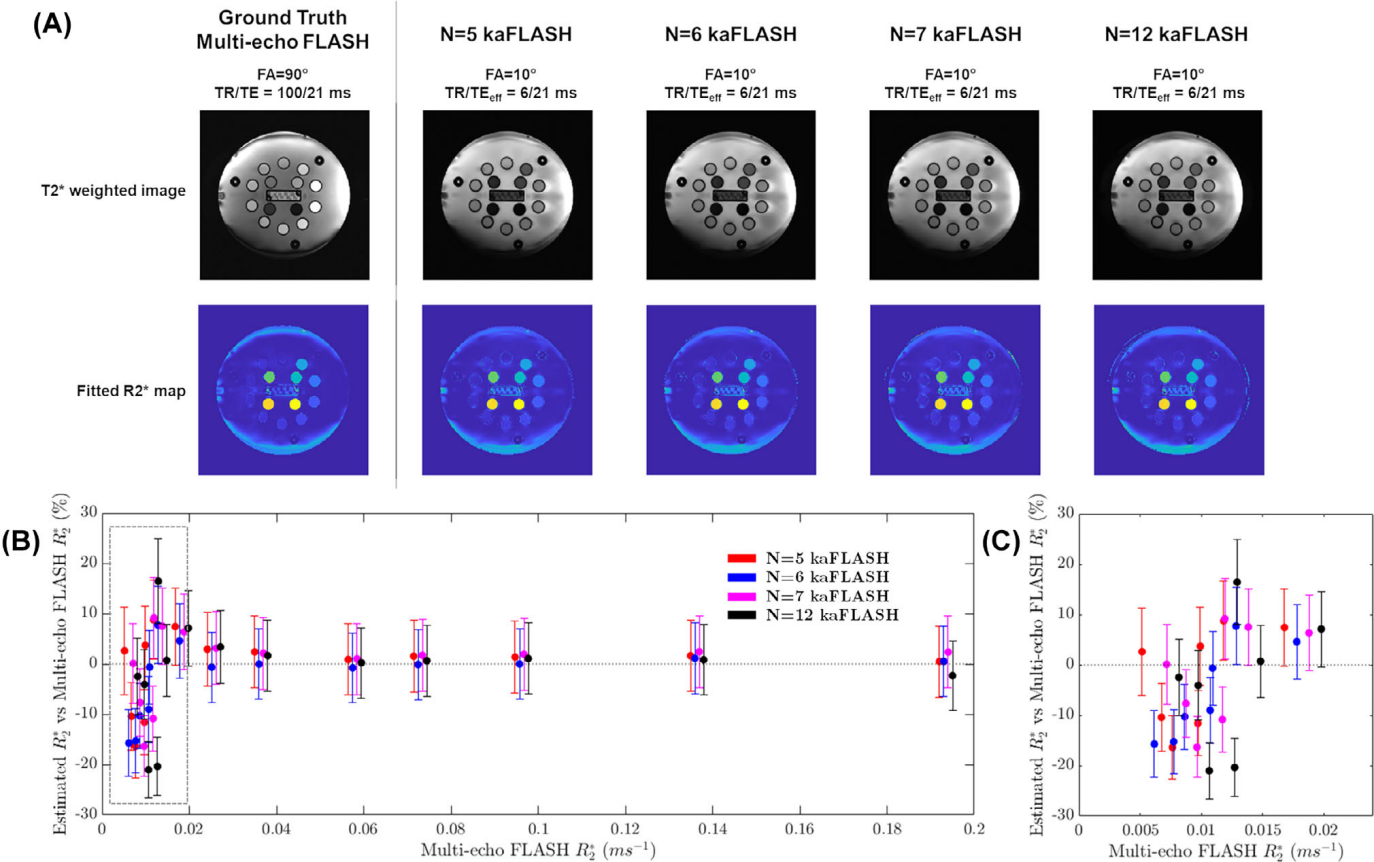
(A) Representative T_2_* weighted images from the phantom experiment at equivalent effective echo times, and the fitted R_2_* maps across the whole object from the first 5 echoes (S_0_–S_4_). (B) Bland–Altman style plot showing percentage deviation from the “ground truth” R_2_* value from multi‐echo FLASH. The four datasets are slightly offset from each other along the *x* axis for visual clarity. kaFLASH and FLASH show close agreement in physiological ranges of interest (R_2_*>0.02 ms^−1^). (C) Zoom in on (B) for R_2_* < 0.02 ms^−1^.

Exemplar T_2_* weighted images and fitted R_2_* maps are shown in Figure 4A. The fitted R_2_* in individual spheres for each approach is given in Figure 4B in comparison to the ground truth multi‐echo FLASH sequence, with error bars indicating the SD in fitted values in a region of interest covering each sphere. For all kaFLASH experiments, R_2_* estimates closely follow those of an equivalent multi‐echo FLASH, apart from small R_2_* (where T_2_* ≫ TR). In these cases, the T_2_*‐weighted kaFLASH images in Figure 4A display areas of heterogeneity (e.g., the main body of the phantom) consistent with the presence of eddy currents, perhaps explaining the bias observed at small R_2_*. Some ringing is evident in the kaFLASH images because of the naïve partial Fourier reconstruction.

### 4.2 SNR efficiency comparisons

**FIGURE 5:**
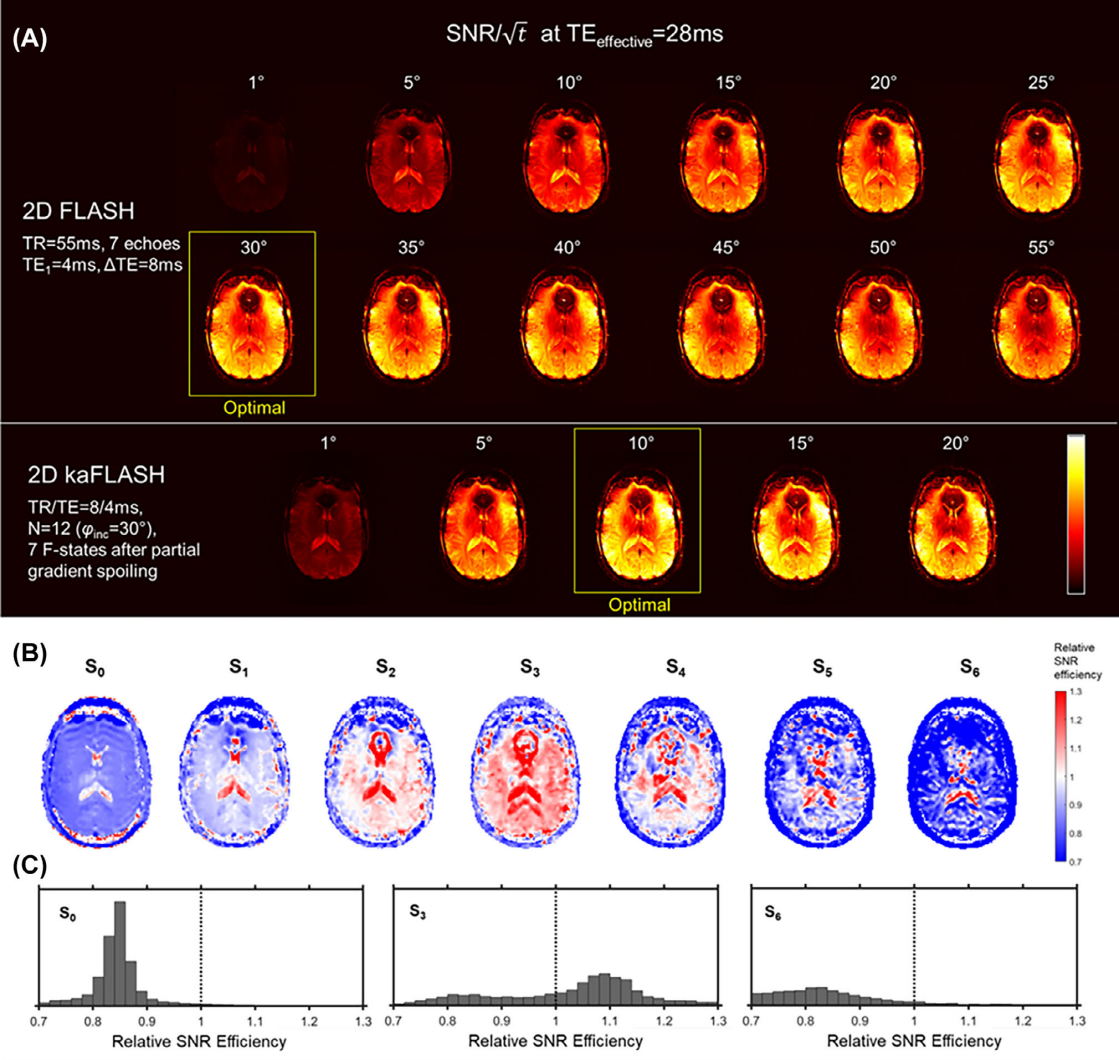
(A) SNR efficiency comparison for a 2D multi‐echo FLASH and equivalent 2D kaFLASH experiment at different flip angles (SNR in arbitrary units, but scaled consistently). (B) Direct SNR efficiency ratio of the two acquisitions at the optimal flip angles (30° for multi‐echo FLASH, 10° for kaFLASH, normalizing by the square root of acquisition time). (C) Histograms of SNR efficiency ratio for S_0_, S_3_, and S_6_. The central echo of kaFLASH has higher SNR from averaging, but other echoes have relatively poorer SNR because of asymmetric sampling.

An SNR efficiency comparison between FLASH and kaFLASH is shown in Figure 5 for the single‐slice 2D experiment with different nominal flip angles, comparing the fully sampled kaFLASH S_3_ signal with the equivalent FLASH echo (TE = 28 ms). kaFLASH is more SNR efficient at the optimal flip angle (10°) compared to the equivalent optimal long TR FLASH (30°). Figure 5 shows a voxelwise SNR efficiency comparison at the optimal flip angles for FLASH (30°) and kaFLASH (10°) across each of the seven echoes, now incorporating the effect of partial Fourier sampling of kaFLASH. This reduces the relative SNR efficiency of kaFLASH for non‐central echoes in the series.

**FIGURE 6:**
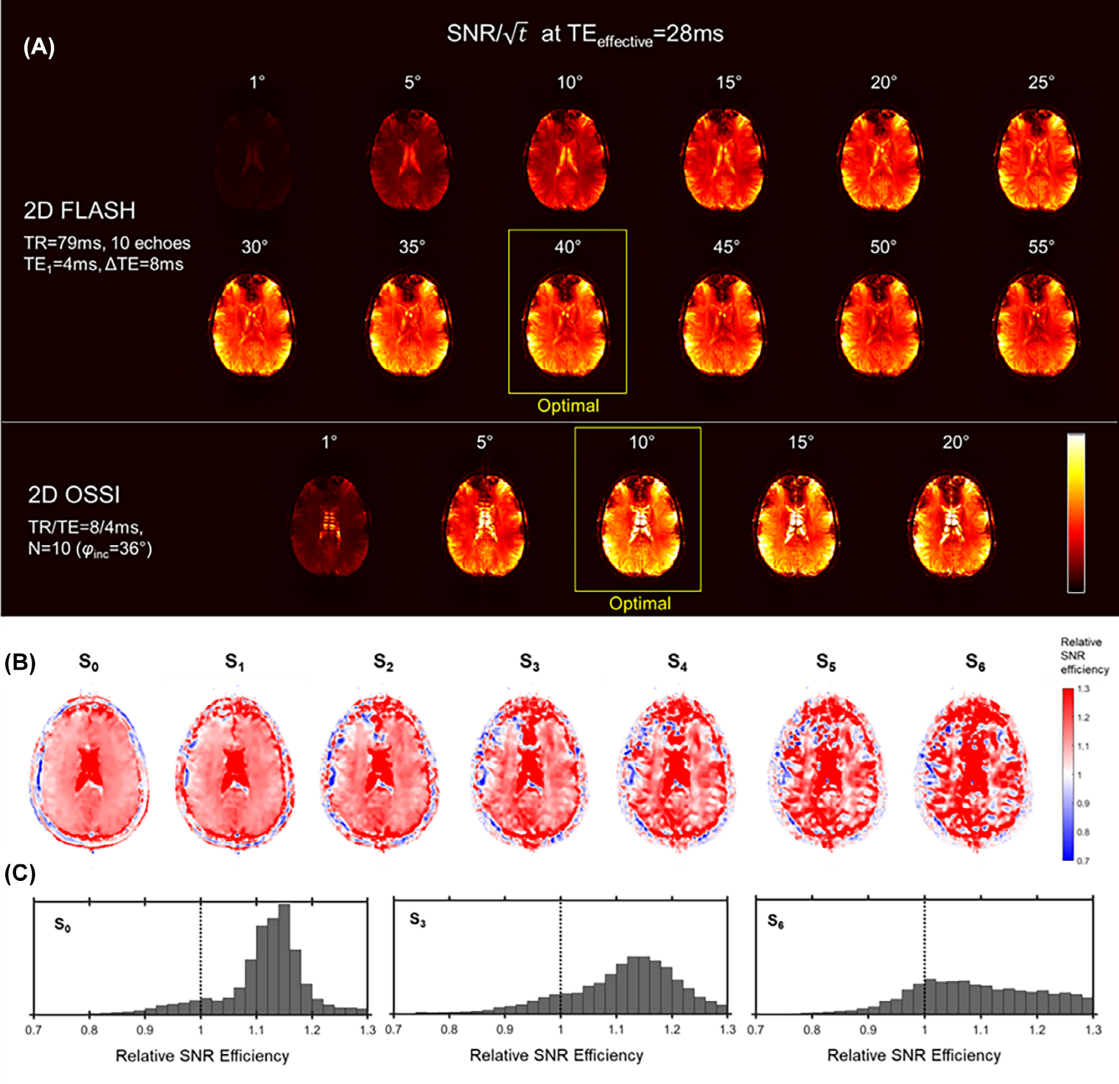
(A) SNR efficiency comparison for a 2D multi‐echo FLASH and equivalent 2D oscillating steady‐state imaging (OSSI) experiment at different flip angles (SNR in arbitrary units, but scaled consistently). (B) Direct SNR efficiency comparison of the two acquisitions at the optimal flip angles (40° for multi‐echo FLASH, 10° for OSSI). (C) Histograms of SNR efficiency ratio for S_0_, S_3_, and S_6_. OSSI shows distinct SNR efficiency improvements over FLASH.

An equivalent SNR efficiency comparison between FLASH and OSSI is shown in Figure 6. In the absence of gradient spoiling, all signals are sampled symmetrically and no partial Fourier reconstruction is required. As a result, there is no additional SNR efficiency penalty for earlier and later echoes in OSSI compared to FLASH. In fact, OSSI has an SNR efficiency improvement of >10% in most brain tissue at the optimal flip angles (30° for FLASH and 10° for OSSI, respectively) across all echoes. There are residual banding artifacts in CSF, where *N* is not large enough to account for all constituent F‐state components.

Theoretically, the optimal flip angle to maximize SNR for each approach is given by the Ernst angle, which is approximately 50% of that deduced for each approach empirically by comparing SNR across nominal flip angles (5° for gray matter in kaFLASH and OSSI, 14°–16° for gray matter in FLASH). This likely reflects the B_1_^+^ inhomogeneity of the 1T× coil at 7 T, such that the delivered flip angle is underestimated compared to the nominal value.

Assuming the Ernst angle for all approaches, and calculating relative SNR efficiency based on Eq. (1), the approaches should all be equally SNR efficient in gray matter, white matter, and CSF to within 2%. However, the SNR from free fluid in the ventricles is substantially enhanced in both kaFLASH and OSSI approaches.

### 4.3 R_2_ * accuracy comparisons

**FIGURE 7:**
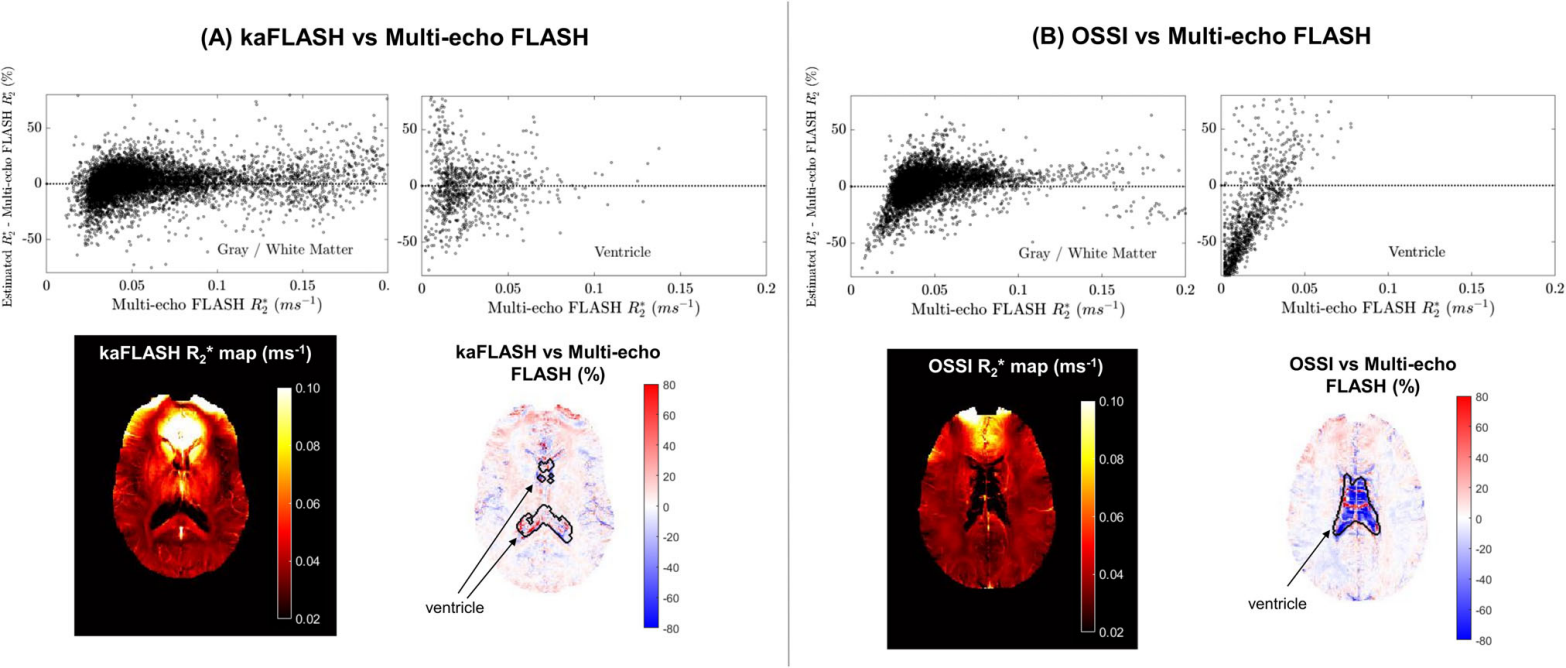
Comparison of R_2_* maps obtained by (A) kaFLASH and (B) oscillating steady‐state imaging (OSSI), each compared to the ground truth R_2_* maps obtained from multi‐echo FLASH, separated into intra‐ and extra‐ventricular regions. OSSI demonstrates bias in regions with low R_2_* such as the ventricles because of the presence of aliasing from high order F‐state signal components.

Figure 7 shows a comparison between each of kaFLASH and OSSI with the ground truth multi‐echo FLASH R_2_* measurements in vivo, with optimal flip angles used for each. In regions with short R_2_*, there is significant underestimation of R_2_* methods with OSSI, as aliasing from higher order F‐states is not incorporated into the fitting model. Note that the high R_2_* anteriorly in either sets of maps is not a fitting error or artifact—it reflects the increased B_0_ inhomogeneity present in these regions of the brain.

### 4.4 Brainstem imaging

**FIGURE 8:**
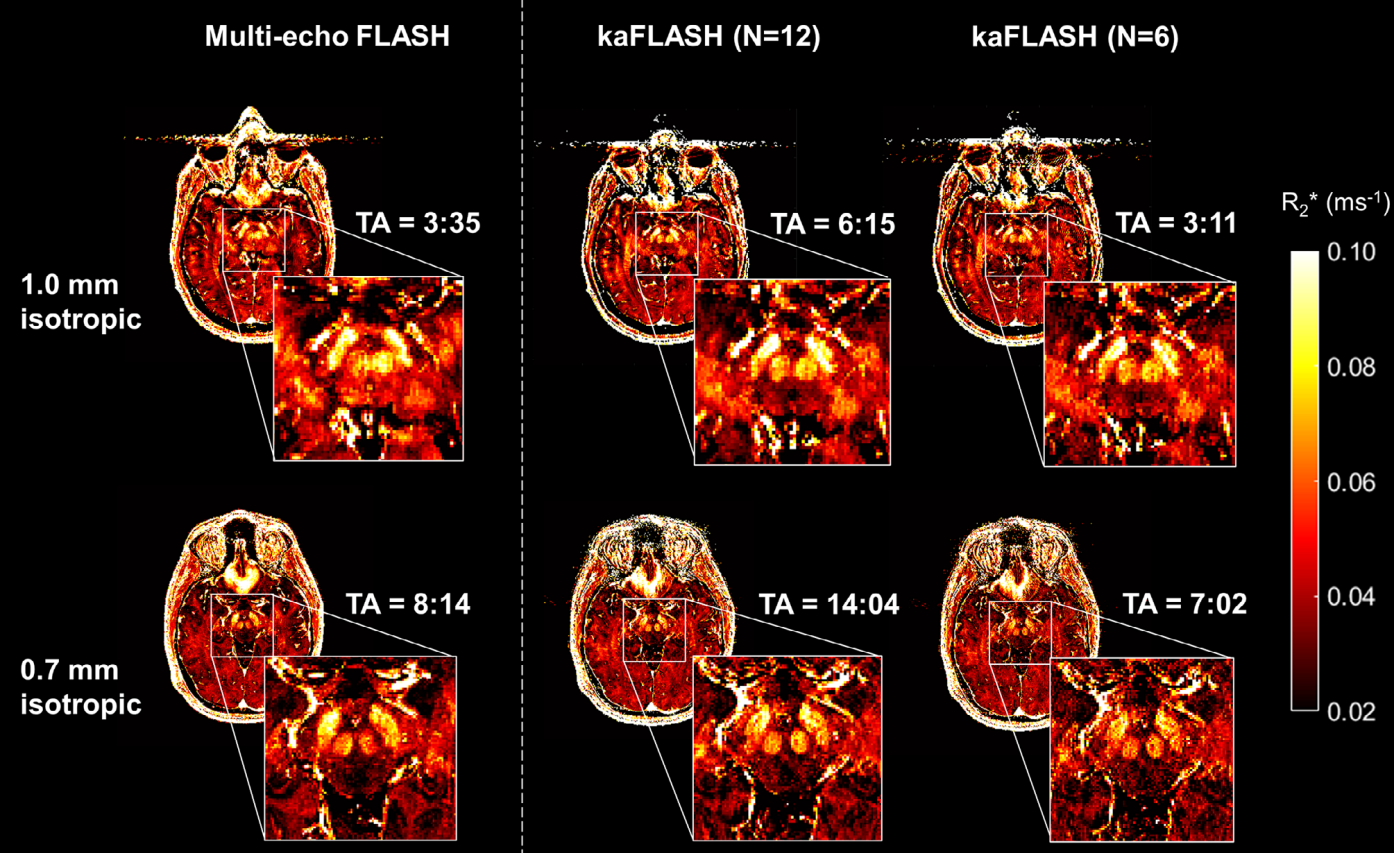
Comparison of R_2_* maps acquired at different spatial resolutions for both multi‐echo FLASH and kaFLASH, showing close agreement between approaches. Acquisition times (TA) also given in min:S.

Figure 8 shows reconstructed 3D data, highlighting iron‐rich midbrain structures. Fitted R_2_* maps from kaFLASH closely resemble those obtained from the multi‐echo FLASH acquisition.

## 5 DISCUSSION AND CONCLUSIONS

In this work, we have described an alternative approach to multi‐echo FLASH sequences widely used for relaxometry. The proposed approach measures all echoes simultaneously during every readout and separates them by modeling their phase evolution under quadratic RF phase cycling. In the absence of gradient spoiling, this increased sensitivity gives SNR advantages over FLASH and allows for more rapid temporal sampling of each echo. If spoiler gradients are used to isolate fewer signal components there is an additional SNR penalty because of asymmetric sampling and partial Fourier reconstruction.

OSSI was introduced as an SNR efficient approach for T_2_*‐weighted imaging, but the fluctuating signal under quadratic RF phase cycling was treated as a nuisance regressor in fMRI experiments. Here, we show that this same fluctuating signal can be decomposed into distinct T_2_*‐weighted signal components with a clear analytical form. This in turn allows for the fluctuating signal to be used for SNR efficient R_2_* relaxometry experiments simultaneously alongside functional imaging.

Because the RF phase increment can be modified explicitly in the pulse sequence, the phase evolution of different signal components can be tightly controlled throughout an experiment and built into the simple combined signal model in Eq. (4). Over a series of *N* TRs, the phase evolution of *N* states (i.e., each row of the matrix in Eq. [5]) is orthogonal. Assuming that any contribution from higher order F‐states is negligible, this allows for clean separation of the *N* signals while retaining the SNR advantages of simultaneously detecting all signals of interest in every readout.

Because each phase encoding line is repeatedly sampled over a timescale of *N* TRs in this work, the experiment is robust to longer term phase effects such as drift in scanner center frequency, which otherwise affect quantitative bSSFP experiments requiring several consecutive acquisitions with distinct steady states.^21^ This repeated sampling also results in signal averaging, which we hypothesize will make it more robust to TR‐to‐TR phase fluctuations in comparison to a multi‐echo FLASH, where each line is sampled once for each contrast resulting in poorer temporal resolution. In theory, the rapid repeated sampling of a given phase encoding line could be used to directly measure dynamic process across for example, the cardiac cycle, and perhaps provide more effective suppression of periodic phase fluctuations from flowing blood from TR‐to‐TR. This would provide distinct advantages in regions like the brainstem, where T_2_*‐weighted imaging is prone to severe motion artifacts.^22^

One limitation of the proposed kaFLASH approach is the asymmetric sampling of different echoes, which leads to ringing artifacts with the naïve partial‐Fourier reconstruction applied in this work. Here, we have used simple signal processing approaches to ensure transparent comparisons with a traditional FLASH approach. However, this is an underestimation of the potential of this approach as there is substantial redundancy in the acquired data, which we have not explored. A combined reconstruction across all echoes would allow considerable acceleration by exploiting shared spatial features or low‐rank signal subspaces, as already applied in OSSI,^23^ as well as other rapid multi‐contrast and asymmetric sampling schemes.^23^, ^24^, ^25^, ^26^

The asymmetric sampling would also be advantageous for super‐resolution experiments,^19^, ^27^ since different overlapping spatial frequency components are sampled from each of the shifted signals, together covering a range up to 2k_max_. Additional components shifted beyond k_max_ could also be reconstructed to provide information, though not directly visible, covering spatial frequencies >2k_max_, allowing for even more spatial resolution enhancement.^19^ Any data corresponding to spatial frequencies beyond k_max_ have been simply discarded in the current work to enable fair comparisons to FLASH by sampling the same spatial frequencies, which also reduces the resultant SNR according to Eq. (7). This has meant discarding nearly 50% of the data for signals at the edge of the acquired k‐space. If this spatial frequency mismatch is ignored and no data is discarded from the kaFLASH acquisition (as may be used in, e.g., a joint reconstruction approach at a higher spatial resolution), both OSSI and kaFLASH have the same SNR benefit from averaging signals over multiple readouts.

Another interesting possibility is to exploit the per‐TR phase term in Eq. (5) to manipulate the spatial encoding process in a way that is distinct for each F‐state component, for example, by producing distinct spatial shifts to separate them within the field of view.^28^

If the OSSI acquisition approach is used (i.e., there is no unbalanced gradient in the TR and all signal components are sampled at the center of k‐space), we can exploit the known phase modulation to obtain a clear SNR advantage without the drawback of asymmetric sampling (Figure 6). However, in this approach *N* must be large enough to cover all non‐zero signal components that contribute to the measured signal (i.e., N.TR >5 T_2_*), to avoid aliasing from signals in higher order echo pathways. This substantially increases the required acquisition time in tissues with long T_2_*, but this would only be a limiting factor in tissues where there is a large volume of free fluid (e.g., CSF in Figure 7).

Another limitation is the potential for increased eddy currents because of the short TRs and rewinding gradients along all axes, along with the additional spoiler gradient. We combined the additional spoiler area with the phase encoding rewinder (offsetting each value by a set amount) to reduce the impact of eddy currents as much as possible. However, in the readout direction where rewinder gradients are necessarily large throughout, the eddy currents should be similar to a multi‐echo FLASH with flyback readout gradients and the same echo spacing.

One interesting feature of the proposed approach is the enhanced brightness of fluid at the optimal lower flip angle for kaFLASH and OSSI. This suggests that these approaches may be more sensitive to BOLD effects if applied in an fMRI context, as proposed previously.^12^

This work only examines the low flip angle regime in which the F‐state signals correspond to different effective TEs in a T_2_* decay curve with a simple analytic form given in Eq. (1). However, this approach could equally be applied to simultaneously measure multiple echoes at higher flip angles and lower *N*, where the relative F‐state signal intensities would give information about T_1_ and T_2_ in a similar way to existing multi‐echo SSFP techniques for example, DESS,^29^ TESS,^30^ or MIRACLE.^31^

In conclusion, we have presented an alternative approach to multi‐echo FLASH sequences, which exploits quadratic RF phase cycling in SSFP acquisitions. Any data already acquired with quadratic phase cycling (e.g., OSSI) can now be re‐examined as an R_2_* relaxometry experiment, enabling both rapid functional contrast and dynamic quantitative R_2_* estimates. The simultaneous measurement of multiple T_2_* weighted signals during every readout and inherent signal averaging effects allow for improved SNR efficiency, with the potential for directly measuring rapid dynamic processes, which would usually corrupt phase information.

## Data Availability

An interactive tutorial about simulating, visualizing and modeling N‐periodic behavior is provided in a Jupyter notebook at https://github.com/petelally/nperiodic_tutorial. Raw data (.dat) files and MATLAB scripts to reproduce phantom experiments are provided at https://github.com/petelally/nperiodic_analysis.

## References

[1] Lewis LD , Setsompop K , Rosen BR , Polimeni JR . Fast fMRI can detect oscillatory neural activity in humans. Proc Natl Acad Sci U S A. 2016;113:E6679‐E6685. 27729529 10.1073/pnas.1608117113 PMC5087037

[2] Chen JE , Glover GH , Fultz NE , Rosen BR , Polimeni JR , Lewis LD . Investigating mechanisms of fast BOLD responses: the effects of stimulus intensity and of spatial heterogeneity of hemodynamics. Neuroimage. 2021;245:118658. 34656783 10.1016/j.neuroimage.2021.118658 PMC8815416

[3] Dowdle LT , Ghose G , Chen CCC , Ugurbil K , Yacoub E , Vizioli L . Statistical power or more precise insights into neuro‐temporal dynamics? Assessing the benefits of rapid temporal sampling in fMRI. Prog Neurobiol. 2021;207:102171. 34492308 10.1016/j.pneurobio.2021.102171 PMC8688272

[4] Chen JE , Polimeni JR , Bollmann S , Glover GH . On the analysis of rapidly sampled fMRI data. Neuroimage. 2019;188:807‐820. 30735828 10.1016/j.neuroimage.2019.02.008 PMC6984348

[5] Agrawal U , Brown EN , Lewis LD . Model‐based physiological noise removal in fast fMRI. Neuroimage. 2020;205:116231. 31589991 10.1016/j.neuroimage.2019.116231 PMC6911832

[6] Liu G , Sobering G , Olson AW , van Gelderen P , Moonen CTW . Fast echo‐shifted gradient‐recalled MRI: combining a short repetition time with variable T_2_* weighting. Magn Reson Med. 1993;30:68‐75. 8371677 10.1002/mrm.1910300111

[7] Chang WT , Nummenmaa A , Witzel T , et al. Whole‐head rapid fMRI acquisition using echo‐shifted magnetic resonance inverse imaging. Neuroimage. 2013;78:325‐338. 23563228 10.1016/j.neuroimage.2013.03.040 PMC3672248

[8] Gibson A , Peters AM , Bowtell R . Echo‐shifted multislice EPI for high‐speed fMRI. Magn Reson Imaging. 2006;24:433‐442. 16677950 10.1016/j.mri.2005.12.030

[9] Sun K , Chen Z , Dan G , et al. Three‐dimensional echo‐shifted EPI with simultaneous blip‐up and blip‐down acquisitions for correcting geometric distortion. Magn Reson Med. 2023;90:2375‐2387. 37667533 10.1002/mrm.29828 PMC10903279

[10] Voit D , Frahm J . Echo train shifted multi‐echo FLASH for functional MRI of the human brain at ultra‐high spatial resolution. NMR Biomed. 2005;18:481‐488. 16292740 10.1002/nbm.998

[11] Boyacioğlu R , Schulz J , Norris DG . Multiband echo‐shifted echo planar imaging. Magn Reson Med. 2017;77:1981‐1986. 27297682 10.1002/mrm.26289

[12] Guo S , Noll DC . Oscillating steady‐state imaging (OSSI): a novel method for functional MRI. Magn Reson Med. 2020;84:698‐712. 31912574 10.1002/mrm.28156 PMC7180136

[13] Foxall DL . Frequency‐modulated steady‐state free precession imaging. Magn Reson Med. 2002;48:502‐508. 12210915 10.1002/mrm.10225

[14] Ganter C . Steady state of echo–shifted sequences with radiofrequency phase cycling. Magn Reson Med. 2006;56:923‐926. 16892200 10.1002/mrm.21010

[15] Vasanawala SS , Pauly JM , Nishimura DG . Fluctuating equilibrium MRI. Magn Reson Med. 1999;42:876‐883. 10542345 10.1002/(sici)1522-2594(199911)42:5<876::aid-mrm6>3.0.co;2-z

[16] Zur Y , Wood ML , Neuringer LJ . Spoiling of transverse magnetization in steady‐state sequences. Magn Reson Med. 1991;21:251‐263. 1745124 10.1002/mrm.1910210210

[17] Weigel M . Extended phase graphs: dephasing, RF pulses, and echoes ‐ pure and simple. J Magn Reson Imaging. 2015;41:266‐295. 24737382 10.1002/jmri.24619

[18] Zur Y , Wood ML , Neuringer LJ . Motion‐insensitive, steady‐state free precession imaging. Magn Reson Med. 1990;16:444‐459. 2077335 10.1002/mrm.1910160311

[19] Lally PJ , Matthews PM , Bangerter NK . Unbalanced SSFP for super‐resolution in MRI. Magn Reson Med. 2021;85:2477‐2489. 33201538 10.1002/mrm.28593 PMC8972796

[20] Lally PJ , Statton B , Matthews PM , Chiew M , Miller KL , Bangerter NK . SNR‐efficient SSFP with k‐space aliasing. Proceedings of the International Society for Magnetic Resonance in Medicine. International Society for Magnetic Resonance in Medicine; 2022.

[21] Shcherbakova Y , van den Berg CAT , Moonen CTW , Bartels LW . Investigation of the influence of B_0_ drift on the performance of the PLANET method and an algorithm for drift correction. Magn Reson Med. 2019;82:1725‐1740. 31317584 10.1002/mrm.27860 PMC6772029

[22] Schwarz ST , Mougin O , Xing Y , et al. Parkinson's disease related signal change in the nigrosomes 1‐5 and the substantia nigra using T_2_* weighted 7T MRI. Neuroimage Clin. 2018;19:683‐689. 29872633 10.1016/j.nicl.2018.05.027 PMC5986169

[23] Guo S , Fessler JA , Noll DC . High‐resolution oscillating steady‐state fMRI using patch‐tensor low‐rank reconstruction. IEEE Trans Med Imaging. 2020;39:4357‐4368. 32809938 10.1109/TMI.2020.3017450 PMC7751316

[24] Dong Z , Wald LL , Polimeni JR , Wang F . Single‐shot echo planar time‐resolved imaging for multi‐echo functional MRI and distortion‐free diffusion imaging. Magn Reson Med. 2025;93:993‐1013. 39428674 10.1002/mrm.30327 PMC11680730

[25] Zhang T , Pauly JM , Levesque IR . Accelerating parameter mapping with a locally low rank constraint. Magn Reson Med. 2015;73:655‐661. 24500817 10.1002/mrm.25161 PMC4122652

[26] Tamir JI , Uecker M , Chen W , et al. T_2_ shuffling: sharp, multicontrast, volumetric fast spin‐echo imaging. Magn Reson Med. 2017;77:180‐195. 26786745 10.1002/mrm.26102 PMC4990508

[27] Hennel F , Tian R , Engel M , Pruessmann KP . In‐plane “superresolution” MRI with phaseless sub‐pixel encoding. Magn Reson Med. 2018;80:2384‐2392. 29656440 10.1002/mrm.27209

[28] Beitone C , Chiew M , Miller KL , Bangerter NK , Lally PJ . Parallel contrast imaging from multi‐Echo SSFP. Proceedings of the International Society for Magnetic Resonance in Medicine. International Society for Magnetic Resonance in Medicine; 2024.

[29] Sveinsson B , Chaudhari AS , Gold GE , Hargreaves BA . A simple analytic method for estimating T_2_ in the knee from DESS. Magn Reson Imaging. 2017;38:63‐70. 28017730 10.1016/j.mri.2016.12.018 PMC5360502

[30] Heule R , Ganter C , Bieri O . Triple echo steady‐state (TESS) relaxometry. Magn Reson Med. 2014;71:230‐237. 23553949 10.1002/mrm.24659

[31] Nguyen D , Bieri O . Motion‐insensitive rapid configuration relaxometry. Magn Reson Med. 2017;78:518‐526. 27605508 10.1002/mrm.26384

